# Is There Any Relationship Between Biochemical Indices and Anthropometric Measurements With Dorsolateral Prefrontal Cortex Activation Among Older Adults With Mild Cognitive Impairment?

**DOI:** 10.3389/fnhum.2021.765451

**Published:** 2022-01-03

**Authors:** Yee Xing You, Suzana Shahar, Mazlyfarina Mohamad, Nor Fadilah Rajab, Normah Che Din, Hui Jin Lau, Hamzaini Abdul Hamid

**Affiliations:** ^1^Dietetics Program and Center for Healthy Aging and Wellness (H-Care), Faculty of Health Sciences, Universiti Kebangsaan Malaysia, Kuala Lumpur, Malaysia; ^2^Diagnostic Imaging and Radiotherapy Program, Faculty of Health Sciences, Universiti Kebangsaan Malaysia, Kuala Lumpur, Malaysia; ^3^Biomedical Sciences Program and Center for Healthy Aging and Wellness (H-Care), Faculty of Health Sciences, Universiti Kebangsaan Malaysia, Kuala Lumpur, Malaysia; ^4^Health Psychology Program, Centre of Rehabilitation and Special Needs, Faculty of Health Sciences, Universiti Kebangsaan Malaysia, Kuala Lumpur, Malaysia; ^5^Nutritional Sciences Program and Center for Healthy Aging and Wellness (H-Care), Faculty of Health Sciences, Universiti Kebangsaan Malaysia, Kuala Lumpur, Malaysia; ^6^Department of Radiology, Faculty of Medicine, Universiti Kebangsaan Malaysia Medical Center, Kuala Lumpur, Malaysia

**Keywords:** anthropometry, biochemical, biomarkers, brain activation, cognitive

## Abstract

Working memory is developed in one region of the brain called the dorsolateral prefrontal cortex (DLPFC). The dysfunction of this region leads to synaptic neuroplasticity impairment. It has been reported that several biochemical parameters and anthropometric measurements play a vital role in cognition and brain health. This study aimed to investigate the relationships between cognitive function, serum biochemical profile, and anthropometric measurements using DLPFC activation. A cross-sectional study was conducted among 35 older adults (≥60 years) who experienced mild cognitive impairment (MCI). For this purpose, we distributed a comprehensive interview-based questionnaire for collecting sociodemographic information from the participants and conducting cognitive tests. Anthropometric values were measured, and fasting blood specimens were collected. We investigated their brain activation using the task-based functional MRI (fMRI; N-back), specifically in the DLPFC region. Positive relationships were observed between brain-derived neurotrophic factor (BDNF) (β = 0.494, *p* < 0.01) and Mini-Mental State Examination (MMSE) (β = 0.698, *p* < 0.01); however, negative relationships were observed between serum triglyceride (β = −0.402, *p* < 0.05) and serum malondialdehyde (MDA) (β = −0.326, *p* < 0.05) with right DLPFC activation (*R*^2^ = 0.512) while the participants performed 1-back task after adjustments for age, gender, and years of education. In conclusion, higher serum triglycerides, higher oxidative stress, and lower neurotrophic factor were associated with lower right DLPFC activation among older adults with MCI. A further investigation needs to be carried out to understand the causal-effect mechanisms of the significant parameters and the DLPFC activation so that better intervention strategies can be developed for reducing the risk of irreversible neurodegenerative diseases among older adults with MCI.

## Introduction

Functional MRI (fMRI) is a noninvasive process that can be used for measuring brain activities since it detects changes related to blood flow (Wright and Wise, 2018). fMRI is regarded as an important tool that helped in detecting the changes that took place in the neural mechanisms of older adults (Belleville and Bherer, 2012). This technique includes several features, which could be used as effective surrogate markers for investigating the cognitive status among older adults (Belleville and Bherer, 2012; Clément and Belleville, 2012). The human brain includes a region called the dorsolateral prefrontal cortex (DLPFC). This region is located in the middle frontal gyrus of the human brain, which is a part of the lateral region in Brodmann’s area 9 and 46 (Barbey et al., 2013). Impaired synaptic neuroplasticity occurs due to a DLPFC dysfunction (Kumar et al., 2017). At present, the site that was most frequently targeted among older adults with mild cognitive impairment (MCI) was the DLPFC as reported in recent studies, which is important for working memory (Wang et al., 2014; Badhwar et al., 2017; Taylor et al., 2019).

Several risk factors have been identified, which indicate cognitive declines such as increased age of adults, inadequate nutrient intake, low educational level, presence of comorbidities, and a lack of physical and social activities (Sachdev et al., 2013; Baumgart et al., 2015; Boyle et al., 2016; Kobe et al., 2016). Additionally, a higher body mass index (BMI) was also associated with a poor cognitive status. Previous studies have also reported that the greater BMI and higher body fat percentage were closely related to poor cognitive status among older adults (Malek Rivan et al., 2019). Cognitive deterioration was also associated with the biochemical profiles of the participants such as the serum lipid profiles. Current evidence suggests that lipids help in regulating the neural functions in the central nervous system since they participate in many local mechanisms associated with the systemic lipid metabolism (Weinstock-Guttman et al., 2011; Hottman et al., 2014). A previous Malaysian study reported that hypertriglyceridemia was related to an increased risk of poor cognitive among older adults with cognitive impairment (Rivan et al., 2020). Although previous studies have focused on the relationship between these parameters with brain activities, however, not all parameters were systematically included in one study among older adults with MCI.

In addition, Revel et al. (2015) reported that oxidative stress-related damage would accelerate the aging process and lead to an age-related cognitive decline. Malondialdehyde (MDA) was observed to be an important biomarker of the lipid peroxidation process, which plays a vital role in progressing dementia (García-Blanco et al., 2017; He et al., 2017; Luo et al., 2020). Age-related oxidative brain damage was significantly increased by lipid peroxidation products, protein oxidation mechanisms, and the oxidative changes that take place in the mitochondrial and nuclear DNA (Zabel et al., 2018). All these factors can lead to irreversible neurodegenerative diseases (García-Blanco et al., 2017; Luo et al., 2020). It is necessary to identify possible biomarkers that can serve better in determining their relationship with cognitive function and can potentially be used as a molecular signature for targeted interventions in the future among older adults with MCI so that their condition could be reversed to successful aging.

A majority of the earlier local studies made use of neuropsychological batteries for assessing the cognitive functions and defining the potential predictors, which led to a decrease in cognitive functioning (Vanoh et al., 2017; Rivan et al., 2020; You et al., 2020). The fMRI technique could measure the changes occurring in the blood flow levels in response to some memory challenges. Hence, it helps in understanding the differences in brain activation levels among older adults. At present, several methods were used to predict the progression of MCI, such as fMRI (Lau et al., 2018; Wright and Wise, 2018), and the analysis of biomarkers in the cerebrospinal fluid and peripheral blood (Hermida et al., 2012). In this study, we aimed to determine the relationship between the various biochemical parameters, anthropometric values, and cognitive function with working memory related to the DLPFC function among the older adults with MCI. We hypothesized that there is a relationship between biochemical parameters, anthropometric values, and cognitive function with working memory related to the DLPFC function among the older adults with MCI.

## Materials and Methods

### Study Design

This is a cross-sectional study that involved 35 community-dwelling older adults with MCI aged 60 years and above as recruited involving two cohorts prior to nutritional intervention studies involving local traditional vegetables (You et al., 2021) and herbs (Lau et al., 2020). They were screened for eligibility based on the inclusion and exclusion criteria. This study was approved by the Medical Research and Ethics Committee of the Universiti Kebangsaan Malaysia (UKM; NN-2019-137), and written informed consent was obtained from all the participants prior to data collection. In this study, the inclusion criteria included older adults aged 60 years and above with MCI based on the criteria described by Petersen et al. (2014) and who were able to communicate in Malay, English, Chinese, or Tamil language participated in the study. The criteria described by Petersen et al. (2014) are stated as follows:

(1) Currently not receiving any clinical judgments on dementia.
(2) Have no or very minimal limitations in instrumental activities of daily livings (IADL) with a score of ≤1.5 SD from the mean norm.
(3) Essentially preserved general cognitive functioning by scoring ≥19 in Mini-Mental State Examination (MMSE).
(4) Objective memory impairment with a score of at least 1.5 SD below the mean average in one or more cognitive tests [Rey Auditory Verbal Learning Test (RAVLT) (immediate recall) or Digit Span] (Vanoh et al., 2017).
(5) Subjective memory complaints.

The exclusion criteria were a history of mental health illness (i.e., Alzheimer’s disease, schizophrenia, and history of stroke), score >5 in the Geriatric Depression Scale (GDS), physical disabilities, alcohol and drug users, being claustrophobic, and having internal metallic or electronic implants.

The sample size was calculated using the formula as follows (Hulley et al., 2013):

*n* = $[\frac{\left(Z\alpha +Z\beta \right)}{C}$]^2^ + 3

where *Z*_α_ = 95% CI = 1.96; *Z*_β_ = 80% power = 0.842; *C* = 0.5 × ln[(1+*r*)/(1−*r*)] = 0.61; *r* = correlation coefficient = 0.515 (Lau et al., 2018), and additional dropout 20%; thus, the total sample size was 35 participants.

### Data Collection

Data collection was carried out at the Center of Healthy Aging and Wellness, UKM and the Hospital Canselor Tuanku Muhriz, UKM. The data that were collected included sociodemographic information, self-reported medical condition, anthropometric measurements, biochemical profiles, neuropsychological tests, and fMRI analysis. The participants signed the informed consent prior to the data collection. A total of five trained field-workers from dietetics, nutrition, and biomedical backgrounds joined the data collection. All field-workers were trained by experienced researchers on anthropometric measurements, blood sample collection, and neuropsychological batteries prior to data collection. No pretesting of the questionnaire was conducted as we used validated questionnaires for all parameters.

The anthropometric measurements such as weight, height, waist circumference, and hip circumference were carried out after informed consent was obtained from the participants. All the measurements were carried out according to the standard procedure (Gibson, 1990). Every measurement was repeated two times, and later, the average value was calculated. BMI was calculated to determine the weight status of the participants. BMI was obtained by dividing weight (kg) with (height)^2^ (m^2^). Equipment was calibrated prior to the measurements.

Participants were asked to fast overnight for at least 10 h to collect blood samples. A total of 20 ml peripheral venous blood was collected and stored in an icebox immediately for delivery purpose. All the basic biochemical profile analyses such as fasting blood sugar, lipid profile, liver function test, and renal profile were analyzed at the medical laboratory (Pathlab Malaysia Sdn Bhd). Serum was isolated by centrifugation and stored at −80°C for 1 month before the biomarker analysis was carried out using commercial ELISA kits. The oxidative stress biomarkers (i.e., MDA), inflammatory biomarkers [i.e., inducible nitric oxide synthase (iNOS) and cyclooxygenase-2 (COX-2)], and brain-derived neurotrophic factor (BDNF) were measured.

We utilized four validated neuropsychological batteries [i.e., MMSE (Ibrahim et al., 2009), Digit Span, Digit Symbol (Weshsler, 1997), and RAVLT (Jamaluddin et al., 2009)] in assessing the global cognitive function, working memory, processing speed, and verbal memory of the participants. The scaled Digit Span and Digit Symbol scores were calculated based on the age-specific tables of the manual (Weshsler, 1997).

**FIGURE 1:**
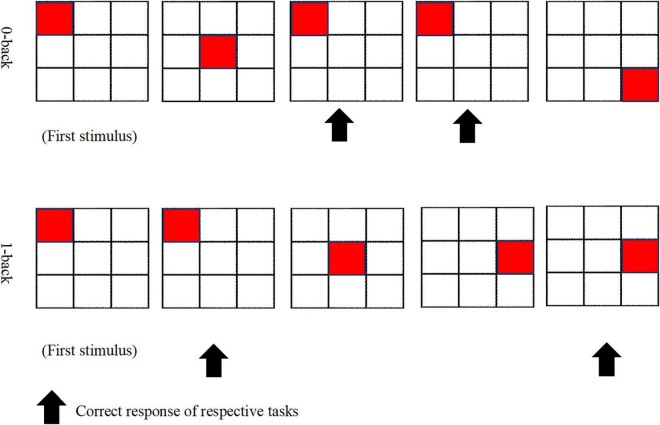
0-back and 1-back paradigms.

A qualified field-worker explained the procedures involved in fulfilling the N-back task. To ensure a clear understanding of the assignment at hand, the participants were provided with a diagram of the task blocks, and the protocol was clearly explained by the field-worker. The two conditions of the N-back task, which were used in this study, had consisted of 0-back and 1-back that were employed by previous studies (Lau et al., 2018; You et al., 2019), which had been created and displayed by using the SuperLab 5 (Cedrus, Los Angeles, CA, United States). N-back holds four blocks for every 0-back and 1-back condition. Figure 1 displays the 0-back and 1-back paradigms. During the 0-back condition, the participants were obliged to react to the stimulus provided and to distinguish if it is similar to the position of the target at the start of the block (i.e., pre-demarcated stimulus). As for the 1-back condition, the participants were called on to decide if the position of the target exhibited is similar to the one previous to it. A radiographer performed a 3-min anatomical scan of the brain, which was followed by approximately 9 min of N-back tasks. The duration of each block was 30 s; there was a 30-s rest between blocks, and the total duration to complete the task was 510 s.

Single-shot spin-echo echo planar imaging (EPI) was obtained; the fMRI data and the fMRI images were performed on a 3.0-tesla magnetic resonance (MR) scanner (MAGNETOM, Trio, Siemens, Erlangen, Germany) with each of the subjects being subjected to a high resolution of T1-weighted anatomical images [repetition time (TR) = 1,900 ms, echo time (TE) = 2.35 ms, voxel dimensions = 1.0 × 1.0 × 1.0 mm, 250 × 250 voxels, 176 slices, slice thickness = 1 mm], while those of the N-back task had been conducted *via* the T2*-weighted imaging data (TR = 3,000 ms, TE = 30 ms, 3-mm isotropic voxels, flip angle = 90°, 27 slices, slice thickness = 4 mm).

The percentage of accuracy and the mean response time (RT) on the N-back task of each participant were then recorded in the calculation of the average data. Correct response (CR) is the percentage of CR from the total response performed by each participant. An index $\frac{\mathrm{Reaction}\mathrm{time}}{\mathrm{Reaction}\mathrm{accuracy}}$ was used to analyze the behavioral performance of the data (You et al., 2019).

### Preprocessing of the Functional Data and First-Level Analysis

The preprocessing and data analysis stage utilized the statistical parametric mapping (SPM12) software that was implemented in MATLAB 9.4.0 R2018a (MathWorks Inc., Natick, MA, United States). The functional images were first slice time corrected followed by realignment. These functional images would then be co-registered to the mean T1-weighted image of the subject and estimated against a standardized Montreal Neurological Institute (MNI) stereotaxic space, where the spatial normalization procedure would involve a 6-parameter affine transformation with a spatial transformation matrix. After undergoing the normalization process, all of the functional volumes were then be subjected to spatial smoothing with a 6-mm full-width half-maximum of isotropic Gaussian kernel as a way of increasing the signal-to-noise level through the removal of the high-frequency information and the reduction of its intersubject variability.

The DLPFC is a key node in the cognitive control network that supports working memory, executive function, attention, planning, and decision-making. Many researchers used the N-back task for evaluating the DLPFC function (Townsend et al., 2010; Diamond, 2013; Lau et al., 2018; You et al., 2019). The DLPFC mask was selected, and it had been defined by the WFU PickAtlas (Maldjian et al., 2003) with Brodmann’s area 9 and 46. Previous studies had identified this volume of interest (VOI) as being responsible for generating the working memory and executive function of the human brain (Townsend et al., 2010; Lau et al., 2018; You et al., 2019). Individual analysis of the participants was performed to determine VOIs within bilateral DLPFC areas to extract averaged percent change of blood oxygen level dependency (BOLD) representing significant activation [*p* < 0.05, family-wise error (FWE) corrected] using MarsBaR toolbox (Brett et al., 2002).

### Statistical Analysis

In this study, the Statistical Package for Social Sciences (SPSS) version 23.0 software was used for carrying out all statistical analyses. The Shapiro–Wilk test was used for determining the data normality (*p* > 0.05). The demographic data of the participants were presented as a percentage value with appropriate SDs. The Pearson’s correlation was used for analyzing the relationship between the demographic characteristics (age, gender, and years of education), biochemical parameters, anthropometric values, and neuropsychological test scores with regard to DLPFC activation (i.e., percent signal change extracted from SPM software). To control for the inflated FWE rates that result from performing multiple tests on the same data, the significance of this partial correlation at a Bonferroni-adjusted alpha level was performed. Furthermore, to make adjustment, the family-wise alpha level (0.05) was divided by the total numbers of variables. In addition, the relationships between significant variables from the Pearson’s correlation analysis and dependent variable (i.e., percent signal change as DLPFC activation) were modeled using the multiple linear regression after adjustments for age, gender, and years of education.

## Results

### Profiles of the Participants

A total of 35 participants comprising of 10 men and 25 women with a mean age of 65 years participated in this study. All the mean values of both the anthropometric measurements and the biochemical parameters were within the normal range except systolic blood pressure, which was higher than the normal values (Table 1).

**TABLE 1:** Demographic characteristics of total participants [expressed in mean ± SD or number (%)].

Parameters	Total participants (*n* = 35)	Reference range ^a,b,c,d,e^
Age (years) ^a^	65.03 ± 3.36	69.5
Gender (men, %) ^a^	10 (28.60)	N/A
Education (years) ^a^	9.26 ± 4.07	5.5
Household income (USD/month) ^a^	402.24 ± 183.43	336
Hypertension (*n*, %) ^b^	15 (42.90)	37%
Type 2 Diabetes Mellitus (*n*, %)^b^	6 (17.10)	41.5%
Hyperlipidemia (*n*, %)^b^	15 (42.90)	60.8%
Height (cm)	156.73 ± 7.83	N/A
Weight (kg)^a^	64.40 ± 10.48	62.4
Body mass index (kg/m^2^)^c^	26.12 ± 3.01	22–27
Waist circumference (cm)^d^	88.33 ± 8.39	<90
Systolic blood pressure (mmHg) ^d^	132.91 ± 16.05	120
Diastolic blood pressure (mmHg)^d^	72.63 ± 10.02	80
Fasting blood glucose (mmol/L)^e^	5.46 ± 1.17	3.9–5.6
Total cholesterol (mmol/L)^e^	5.40 ± 0.96	<5.2
Low density lipoprotein (mmol/L)^e^	3.27 ± 0.90	<2.6
High density lipoprotein (mmol/L)^e^	1.52 ± 0.37	>1.04
Triglyceride (mmol/L)^e^	1.38 ± 0.55	<1.7
Sodium (mmol/L)^e^	141.51 ± 3.06	137–150
Potassium (mmol/L)^e^	4.45 ± 0.52	3.5–5.3
Urea (mmol/L)^e^	4.34 ± 1.11	1.7–8.4
Creatinine (μmol/L)^e^	69.31 ± 18.16	62–115
Uric acid (μmol/L)^e^	0.35 ± 0.01	0.20–0.42
Total protein (g/L)^e^	72.51 ± 6.20	57–82
Albumin (g/L)^e^	43.91 ± 2.38	32–48
Globulin (g/L)^e^	28.63 ± 6.53	20–50
Total bilirubin (μmol/L)^e^	11.86 ± 4.77	3–19
Alkaline phosphatase (U/L)^e^	74.54 ± 17.11	39–117
Alanine aminotransferase (ALT) (U/L)^e^	20.20 ± 11.19	0–40
Aspartate aminotransferase (AST) (U/L)^e^	22.71 ± 5.43	0–40
Inducible nitric oxide synthase (iNOS) (pg/ml)	182.31 ± 29.21	N/A
Cyclooxygenase-2 (COX-2) (ng/ml)	1.34 ± 0.70	N/A
Brain-derived neurotrophic factor (BDNF) (pg/ml)	114.52 ± 53.56	N/A
Malondialdehyde (MDA) (ng/ml)	211.22 ± 50.27	N/A
Neuropsychological batteries		
MMSE ^a^	25.94 ± 2.18	19
Digit Span (Scaled score)^a^	13.14 ± 1.90	7.7
RAVLT immediate recall ^a^	6.83 ± 1.47	7
RAVLT delayed recall^a^	6.11 ± 2.01	6
Digit Symbol (Scaled score)^a^	7.63 ± 2.43	8
fMRI behavioral performance		
Reaction accuracy (N-back) (%)	62.07 ± 10.59	N/A
Reaction time (N-back) (ms)	2245.95 ± 297.87	N/A
RT/RA index	37.35 ± 8.87	N/A
fMRI brain activation		
0-back mean percent signal change (%) Left DLPFC	0.73 ± 0.52	N/A
0-back mean percent signal change (%) Right DLPFC	0.83 ± 0.40	N/A
1-back mean percent signal change (%) Left DLPFC	0.82 ± 0.49	N/A
1-back mean percent signal change (%) Right DLPFC	0.94 ± 0.40	N/A

*^a^Nationwide aging population research in Malaysia (Shahar et al., 2016).*

*^b^National Health and Morbidity Survey 2019 in Malaysia.*

*^c^Body mass index for older adults (Winter et al., 2014).*

*^d^WHO cutoff guidelines (World Health Organisation, 2011).*

*^e^Normal range values from the accredited biomedical laboratory. DLPFC, dorsolateral prefrontal cortex; fMRI, functional MRI; MMSE, Mini-Mental State Examination; RAVLT, Rey Auditory Verbal Learning Test; RA, reaction accuracy; and RT, reaction time.*

### Functional MRI Brain Activation

**FIGURE 2:**
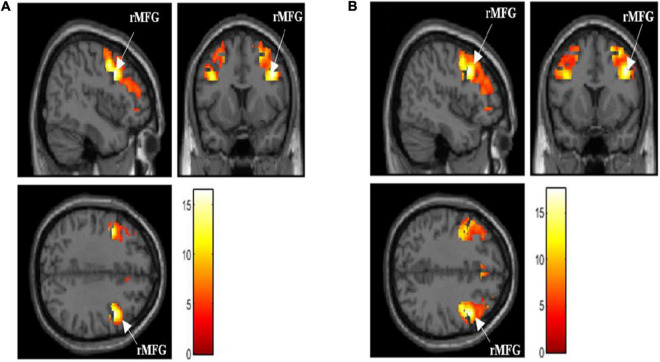
**(A)** Activated brain region in dorsolateral prefrontal cortex (DLPFC) when performing 0-back task [*p* < 0.05, family-wise error (FWE) corrected]. **(B)** Activated brain region in DLPFC when performing 1-back task [*p* < 0.05, FWE corrected]. rMFG, right middle frontal gyrus; activation intensity: Red to White (low to high).

The activated brain region in DLPFC (middle frontal gyrus and Brodmann’s area 9 and 46) when performing the N-back task (*p* < 0.05, FWE corrected) is presented in Table 2. The total voxels activated in DLPFC for 0-back and 1-back being 5,141 and 7,915 voxels, respectively, with the highest activation observed in the right middle frontal gyrus (rMFG) for both 0-back and 1-back tasks (*p* < 0.05, FWE corrected). Figure 2 demonstrates that the middle frontal gyrus, precentral gyrus, superior frontal gyrus, and inferior frontal gyrus were activated while the participants performed 0-back and 1-back tasks.

**TABLE 2:** Activated brain regions during 0-back and 1-back task [*p* < 0.05, family-wise error (FWE) corrected].

Anatomical region	L/R	Coordinates			Voxels activated	Maximum *T*-value
		*x*	*y*	*z*		
**0-back**						
Middle frontal gyrus	R	44	10	14	2,922	16.47
	L	−34	0	50	2,188	14.83
Precentral gyrus	L	−36	4	30	3	10.58
Inferior frontal gyrus	L	−42	42	−2	26	5.31
Superior frontal gyrus	R	4	40	38	2	4.72
**1 back**						
Middle frontal gyrus	R	46	12	38	4,269	17.66
	L	−40	8	34	3,551	16.78
Superior frontal gyrus	R	4	30	38	73	9.14
	L	−6	30	38	18	7.73
Inferior frontal gyrus	R	50	40	2	2	5.89
Precentral gyrus	R	62	6	26	2	4.99

*L, left; R, right.*

### Relationship Between Demographic Characteristics, Anthropometric Measurements, Biochemical Profiles, Biomarkers, Cognitive Tests, and Brain Activation

Table 3 shows the relationship between demographic characteristics, anthropometric measurements, biochemical indices, and cognitive tests with DLPFC. Women participants demonstrated higher right DLPFC activation compared to men participants while performing the 1-back task (*p* < 0.05). Significant positive correlations were observed between years of education (*r* = 0.400, *p* < 0.05), high density lipoprotein (*r* = 0.431, *p* < 0.01), serum BDNF (*r* = 0.407, *p* < 0.0125), MMSE (*r* = 0.466, *p* < 0.01), and RAVLT immediate recall (*r* = 0.451, *p* < 0.01) with right DLPFC activation while the participants performed the 1-back task. However, significant negative correlations were observed between age (*r* = −0.340, *p* < 0.05), serum triglyceride for female (*r* = −0.450, *p* < 0.01), and serum MDA (*r* = −0.455, *p* < 0.0125) with right DLPFC activation while the participants performed the 1-back task. Further analysis using the multivariate linear regression is demonstrated in Table 4. The findings showed positive relationships between BDNF (β = 0.494, *p* < 0.01) and MMSE (β = 0.698, *p* < 0.01); however, negative relationships were observed between serum triglyceride (β = −0.402, *p* < 0.05) and serum MDA (β = −0.326, *p* < 0.05) with right DLPFC activation (*R*^2^ = 0.512) while the participants performed the 1-back task after adjustments for age, gender, and years of education.

**TABLE 3:** Relationship between demographic characteristics, anthropometric measurements, biochemical indices, and cognitive tests with dorsolateral prefrontal cortex (DLPFC) activation (*n* = 35).

		0-back right DLPFC activation	0-back left DLPFC activation	1-back right DLPFC activation	1-back left DLPFC activation

		**Participants (*n* = 35)**	**Participants (*n* = 35)**	**Participants (*n* = 35)**	**Participants (*n* = 35)**
Age ^a^	r	–0.118	–0.031	−0.340*	–0.020
	p	0.500	0.860	0.045	0.908
Gender ^b^	p	0.171	0.630	0.042*	0.629
Education years ^a^	r	0.131	0.107	0.400*	0.119
	p	0.453	0.539	0.017	0.496
Anthropometric measurements ^a^					
Body mass index	r	–0.226	–0.264	–0.166	–0.413
	p	0.479	0.407	0.606	0.182
Biochemical profiles ^c^					
Fasting blood glucose	r	–0.304	–0.010	–0.047	–0.190
	p	0.075	0.954	0.790	0.275
Total cholesterol	r	–0.158	–0.252	–0.022	–0.167
	p	0.365	0.144	0.900	0.337
Low density lipoprotein	r	–0.166	–0.263	–0.121	–0.097
	p	0.340	0.127	0.487	0.579
High density lipoprotein	r	0.111	0.163	0.431*	0.187
	p	0.526	0.349	0.009	0.281
Triglyceride	r	–0.082	–0.176	−0.450*	–0.007
	p	0.638	0.311	0.008	0.967
Biomarkers *^d^*					
Inducible nitric oxide synthase	r	–0.300	–0.281	–0.258	–0.156
	p	0.080	0.102	0.135	0.372
Cyclooxygenase-2	r	–0.120	–0.061	–0.363	–0.006
	p	0.671	0.829	0.183	0.982
Brain-derived neurotrophic factor	r	0.063	0.026	0.407*	0.085
	p	0.718	0.881	0.018	0.629
Malondialdehyde	r	–0.384	–0.270	−0.455*	–0.172
	p	0.023	0.117	0.008	0.323
Neuropsychological batteries ^c^					
Mini-Mental State Examination	r	0.105	0.273	0.466*	0.206
	p	0.548	0.112	0.005	0.236
Digit Span	r	0.114	0.015	0.252	0.067
	p	0.516	0.933	0.144	0.704
RAVLT immediate recall	r	0.291	0.328	0.451*	0.233
	p	0.090	0.055	0.008	0.177
RAVLT delayed recall	r	0.243	0.136	0.105	0.093
	p	0.160	0.436	0.547	0.595
Digit symbol	r	0.081	0.081	0.077	0.086
	p	0.643	0.642	0.659	0.625

Significant at ^a^p < 0.05*, ^c^p < 0.01*, ^d^p < 0.0125* using the Pearson’s correlation after the Bonferroni correction.

Significant at ^b^p < 0.05* using the independent t-test.

*DLPFC, dorsolateral prefrontal cortex; fMRI, functional MRI; and RAVLT, Rey Auditory Verbal Learning Test.*

**TABLE 4:** Multiple linear regression model of biochemical profiles, biomarkers, cognitive tests, and DLPFC activation.

Parameter	1-back right DLPFC activation			
	*R* ^2^	Adjusted odd ratio (95% CI)	*t*	*p*-value
High density lipoprotein	0.512	0.197 (−0.183−0.205)	0.981	0.336
Triglyceride		−0.402 (−0.552 to −0.332)*	–2.301	0.029
Brain-derived neurotrophic factor		0.494 (0.301−0.606)**	2.902	0.007
Malondialdehyde		−0.326 (−0.354 to −0.305)	–2.175	0.038
Mini-Mental State Examination		0.698 (0.591−0.796)**	3.912	0.001
RAVLT immediate recall		0.071 (0.059−0.082)	1.641	0.113

Significant at p < 0.05* and p < 0.01** using multiple linear regression (MLR).

*MLR model was adjusted by age, gender, and years of formal education.*

*DLPFC, dorsolateral prefrontal cortex; RAVLT, Rey Auditory Verbal Learning Test.*

## Discussion

In this study, we have successfully determined the relationship between the various biochemical parameters, anthropometric values, and neuropsychological test scores with the DLPFC activation among the older adults with MCI. The results of our study showed that older participants with MCI had lower DLPFC activation, which was associated with increased lipid peroxidation and oxidative stress. A few earlier studies indicated that lipid peroxidation led to oxidative degradation of the polyunsaturated fatty acids in cells. This could cause a release of many inflammatory and pro-inflammatory factors that promote cell proliferation or apoptosis (Libetta et al., 2011; Redza-Dutordoir and Averill-Bates, 2016). Additionally, the brain shows a higher oxidative metabolism that can lead to the production of a higher concentration of reactive oxygen species (ROS; Redza-Dutordoir and Averill-Bates, 2016; Salim, 2017; You et al., 2018). ROS molecules induce neuronal death and further decrease the activation potential value of the neurons, which could decrease the local demand for the oxygenation process. This, in turn, decreased the blood volume supply or perfusion (Belaïch et al., 2015). Thereafter, the intensity of the BOLD images collected from the activated cortical regions in the brain was decreased. This was based on the fact that community-dwelling older adults showed significant oxidative stress (Belaïch et al., 2015). Thus, it could be concluded that oxidative stress might directly affect brain activation as the ROS molecules were involved in the neurodegenerative metabolic process (Numakawa et al., 2011; Salim, 2017).

Additionally, serum BDNF showed a positive association with right DLPFC activation. Phillips (2017) showed that an adequate BDNF level modulated neuronal plasticity, which helped in maintaining the neuronal functions and promoted the adaptation to the exogenous and endogenous stressors particularly during chronic stress or depression. The strongest evidence for the role of BDNF in cognitive performance comes from the relatively large body of literature using a human model to elucidate the role of BDNF on spatial memory (Erickson et al., 2010; Piepmeier and Etnier, 2015). Correlational evidence with older adults has shown that serum BDNF was associated with hippocampal volume and spatial memory (Piepmeier and Etnier, 2015; Lau et al., 2020). Erickson et al. (2010) utilized MRI, ELISA, and measures of spatial memory to assess the association between age-related decreases in brain volume, BDNF, and memory in older adults. Results indicated that subjects with MCI had significantly lower concentrations of BDNF, smaller hippocampal volumes, and worse performance on spatial memory tasks as compared to successful aging participants (Erickson et al., 2010). In another study, Mattson et al. (2004) stated that the age-dependent impairment in the cognitive functions could be due to a decrease in the BDNF expression in the primary areas of the brain, which were affected by the aging-related issues (Mattson et al., 2004). Thus, it was concluded that BDNF is an important biomarker that was closely associated with brain activation among older adults with MCI.

In this study, we noted a negative relationship between serum triglyceride levels and brain activation. Similar results were reported earlier by Parthasarathy et al. (2017) who observed that the serum triglyceride levels were related to the brain function of the healthy older participants. Hence, in this study, we hypothesized that there could be a significant relationship between the serum triglyceride levels and brain activation in older adults with MCI. The serum triglycerides can pass the blood-brain barrier (BBB; Banks et al., 2018) and can regulate the transport of insulin and gastrointestinal hormones across BBB, which could negatively affect brain activation (Urayama and Banks, 2008; Banks, 2012; Parthasarathy et al., 2017). Hypertriglyceridemia can trigger the production of ROS molecules in the mitochondrial electron system, which causes lipid peroxidation in the cell membranes and leads to the generation of lipid peroxide and other radicals. An increase in the lipid peroxidation mechanism was attributed to oxidative stress and could lead to a cognitive decline (Bradley-Whitman and Lovell, 2015; You et al., 2018). Thus, it was concluded that the actual effect of the serum triglyceride levels on brain activation has not been explained clearly, and further studies need to be carried out to determine their actual relationship with neuron function in the DLPFC.

Furthermore, another highlight of the outcomes of this study is the positive relationship between the MMSE scores and DLPFC activation among participants with MCI. MMSE is a validated neuropsychological test that assesses global cognitive functions (i.e., visuospatial, attention, and executive functions). It is highly sensitive to the functions of the frontal lobe (Ibrahim et al., 2009). DLPFC also plays a vital role in controlling the verbal and working memory, particularly, manipulating the stimuli, integrating all the collected information, selecting the best response while making decisions, and temporarily storing vital information (Barbey et al., 2013; Bosch et al., 2013; Buckholtz et al., 2015; You et al., 2019). These characteristics are supported by the fact that the cognitive functions and the verbal memory were associated with the structure of the white matter tracts related to the DLPFC (Turken et al., 2008).

Some researchers also investigated the association between anthropometric values and cognitive function with brain activation (Papachristou et al., 2015; Won et al., 2017). However, this study did not observe any relationship of these parameters with brain activation. This could be attributed to the activation that may have occurred in different regions in the brain, which was not studied.

In addition, the maximal DLPFC activation was observed in the rMFG region when the participants performed the N-back task. This was attributed to the fact that they showed a right-hemispheric dominance during the visual-spatial processing phase when they performed the N-back task (Pisella et al., 2011). Generally, the prefrontal cortex regions, such as the rMFG, left superior frontal gyrus, and inferior frontal gyrus, control the working memory, attention, and executive functioning in humans (Lara and Wallis, 2015; Koyama et al., 2017). A few earlier studies proved that the middle frontal gyrus was involved in various working memory tasks such as numerical operations or word reading (Koyama et al., 2017; Lau et al., 2018; You et al., 2019).

The strength of this article is the use of the fMRI approach that helped in investigating all underlying changes that affected the cerebral hemodynamic responses to the anthropometric values, biochemical profiles, and blood biomarkers. As fMRI is noninvasive and does not involve the use of ionizing radiation, it is suitable for older adults. The limitation of this study is that the significant findings were not shown in men, probably due to a smaller sample size, as compared to women. In addition, the brain activation was analyzed using the VOI analysis (i.e., DLPFC); however, the whole-brain analysis is recommended to investigate the biochemical and anthropometric variables related to other activated brain regions in the future. We also suggest that a longitudinal study should be conducted to examine the association between the biochemical indices and anthropometric measurements with brain activation and the inclusion of a healthy control group using the whole-brain analysis in future study.

## Conclusion

Abnormal lipid profile as indicated by a higher level of serum triglycerides, oxidative stress, and lipid peroxidation and also as indicated by a higher serum MDA and a lower BDNF was associated with poorer brain activation as assessed using the right DLPFC activation, particularly in women subjects. A further investigation needs to be carried out for understanding the mechanisms affecting the relationships between all the above mentioned parameters and the DLPFC activation so that better intervention strategies can be developed to reduce the risk of irreversible neurodegenerative diseases among older adults with MCI.

## Data Availability Statement

The original contributions presented in the study are included in the article/supplementary material, further inquiries can be directed to the corresponding author.

## Ethics Statement

The studies involving human participants were reviewed and approved by the Medical Research and Ethics Committee of the Universiti Kebangsaan Malaysia (NN-2019-137). The patients/participants provided their written informed consent to participate in this study. Written informed consent was obtained from the individual(s) for the publication of any potentially identifiable images or data included in this article.

## Author Contributions

YXY contributed to the drafting of the manuscript, acquisition and analysis of data, project administration, interpreting and validation of the results, and operations. SS contributed to study conception and design, acquisition and analysis of data, interpreting and validation of the results, and supervision. MM contributed to the design of paradigms and data processing and analysis of data. NFR contributed to interpreting and validation of the results as well as supervision. NCD contributed to the acquisition of data and operations. HJL contributed to interpreting and validation of the results as well as operations. HAH contributed to interpreting and validation of the results. All authors contributed to the manuscript and approved the submitted version.

## Conflict of Interest

The authors declare that the research was conducted in the absence of any commercial or financial relationships that could be construed as a potential conflict of interest.

## Publisher’s Note

All claims expressed in this article are solely those of the authors and do not necessarily represent those of their affiliated organizations, or those of the publisher, the editors and the reviewers. Any product that may be evaluated in this article, or claim that may be made by its manufacturer, is not guaranteed or endorsed by the publisher.
